# No mineralogic or geochemical evidence of impact at Tall el-Hammam, a Middle Bronze Age city in the Jordan Valley near the Dead Sea

**DOI:** 10.1038/s41598-022-08216-x

**Published:** 2022-03-25

**Authors:** Steven J. Jaret, R. Scott Harris

**Affiliations:** 1 Department of Earth and Planetary Sciences, American Museum of Natural History, New York, NY 10024 USA; 2 Department of Space Sciences, Fernbank Science Center, Atlanta, GA 30307 USA

**Keywords:** Meteoritics, Petrology

**arising from**: T. E. Bunch et al.; *Scientific Reports* 10.1038/s41598-021-97778-3 (2021).

In their recent Nature Scientific Reports article, Bunch et al.^1^ present several observations from the geologic and archeologic record and conclude that the destruction of the Bronze Age city Tall el-Hammam was caused by a cosmic air burst. Here, we challenge their mineralogic and geochemical observations. This is important because as determined by the impact cratering community, these are the only kinds of evidence that can provide uniquely diagnostic evidence of impacts or airburst and are the only criteria currently acceptable for confirmation of impact.

Studies of confirmed and doubtful impacts and air bursts that could have affected humans have generated considerable excitement and debate over the last two decades^2–5^. The excitement importantly emphasizes the critical role of geological studies in the field of planetary defense. While we welcome provocative hypotheses that implicate hypervelocity collisions in historic disasters, we must recognize that the implication for assessing future risks demands adherence to the highest burdens of evidence.

## The importance of uniquely diagnostic evidence for shock metamorphism

Bunch et al.^1^ however, fail to demonstrate convincing evidence of impact-shocked minerals in the proposed air burst deposit by not following well-established guidelines and criteria^6–13^. And although the authors also report the presence of ultra-high-temperature mineral phases and geochemical enrichments in melts that often are used to distinguish impact glasses from those produced by volcanoes, lightning, and pyrometamorphism, these criteria have little meaning in the context of archeological debris where our ancestors had access to ceramic and smelting technologies. Much of the melt glass presented in their study is closely associated with pottery; many specimens are pieces of remelted ceramics like one might find in spoils and dumps; and many examples they provide to demonstrate ultra-high temperatures, such as the formation of baddeleyite rims on zircon and the embayment of refractory phases by dissolution in hot melts, are well-documented in ancient slags^14^. We emphasize the importance of uniquely diagnostic criteria for confirming impact or shock events. The impact materials community has outlined several criteria considered unique to impacts, and detailed what kinds of analyses are suitable for making such observations^6–12^. Natural materials are complex, and so it is possible to find non-diagnostic features within bona-fide shocked materials. The converse, however, is not true. The presence of only non-diagnostic features—even if these same features sometimes occur in shock materials—is not sufficient to claim impact. Without unequivocal evidence of shock metamorphism, minerology and geochemistry that cannot be explained by human activities, or definitive remains of an extraterrestrial impactor, the Bunch et al.^1^ claim that the city’s demise was the result of a cosmic catastrophe cannot be supported.

We outline below many of the ways their report fails to meet the standards required to demonstrate an extraterrestrial cause.

### The putative “shocked quartz” grains do not exhibit the attributes required for shocked quartz

Their optical photomicrographs intended to illustrate planar deformation features (PDFs) do not exhibit the properties of PDFs as established by the literature^6–13,15–20^. Specifically, the “lamellae” in the grains are not planar, and instead pinch and swell in thickness, and have irregular spacing. These are in direct contrast to shocked quartz produced in nature, nuclear explosions, and laboratory experiments which have planar morphologies, uniform spacing, and do not change thickness^9,10,12,15,17,19,20^^.^

The authors present measurements of their “lamellae” orientation as evidence of impact origin. This is insufficient and not an accepted method for verifying authenticity of shocked quartz. In shocked quartz, PDFs occur at specific orientations and measuring their orientation is often part of the characterization process^16,21^. However, simply having an orientation that is common to PDFs does not universally demonstrate a shock origin. In the presented dataset, shock lamellae cannot be distinguished from tectonic lamellae based on orientation alone. In fact, all but two of the twelve indexed “lamellae” have orientations common to frequently documented twin planes in tectonically or thermally stressed quartz. Only the (0001), or basal, orientations might be interesting, as bona fide basal Brazil twins generally are believed to be diagnostic of shock. But these are not displayed in the figures despite the author’s emphasizing the (0001) orientation in Fig. 21, which are their comparative “known airburst” grains.

Any informative value the histogram in Fig. 20 might have is further complicated by the fact that it apparently lumps multiple kinds of features (planar fractures (PFs), feather features (FFs), and PDFs) as “shock lamellae.” These features occur under different conditions, have different formation processes, and have different degrees of uniqueness to impact. For example, PDFs are recognized as unique diagnostic effects of shock metamorphism, whereas FFs are believed to be associated with impact-induced shearing, and PFs may form across a wide range of impact and non-impact conditions. Basal PDFs (Brazil twins) and basal PFs can lead to very different conclusions. But from the data presented, we do not have any way to determine which of these features are present in this study. Also, the putative feather features, apparently only one occurrence illustrated in Fig. 18e, do not conform to the definition of feather features. Feather features intersect the associated fracture at acute angles^22^. The faint features shown in Fig. 18e are at right angles to the fracture, a very common microfabric in tectonically deformed quartz.

The authors state that “*TeH grains pass four main tests that confirm impact-shock and contra-indicate tectonic lamellae: (i) they are crystallographically aligned; (ii) some are parallel to the c-axis; (iii) all are visible in SEM imaging and epi-illumination indicating presence of amorphous quartz; and (iv) all are non-luminescent (black) or red under CL imaging.*” None of these are unique tests for shocked quartz, nor are these the criteria established by the impact community for identifying shocked quartz^8,17,18^. Lamellae occurring parallel to the c-axis are common in natural and experimental tectonic deformation which form lamellae parallel to c in response to slip along the (0001) plane^23^. Electron beam imaging (including CL imaging) are frequently used in support of optical microscopy to characterize independently verified PDFs, but cannot alone be seen as diagnostic. Moreover, the CL images in Fig. 18 showing lamellae that—despite being generally planar—occur as thick, discontinuous, widely spaced lenses of variable grayscale precisely match the description of tectonic lamellae provided by Hamers and Drury^24^.

Bunch et al.^1^ also claim that *“SEM analyses of HF-etched grains allow for unambiguous visual distinction between glass-filled PDFs and glass-free tectonic lamellae, which are not visible on the surface of sectioned grains”* as justification that their features are shock-produced. While HF etching can **enhance** the appearance of PDFs, true PDFs are always visible optically without etching. Etching can be problematic because non-shock features such as twin planes, (eg., Dauphine, Brazil, etc.) and lattice dislocations, which otherwise are invisible in optical microscopy will appear when grains are etched by HF^25^. Dauphine twins can result from thermal stress at the alpha–beta quartz transition (573 °C), and by necessity should form in quartz grains encased in high temperature melt. Consequently, the argument that etching uniquely identifies shock features is incorrect.

## Extraterrestrial geochemistry as evidence of impact

### The geochemical data presented is not adequate or sufficient for confirming presence of extraterrestrial material

In addition to petrographic observations of shock metamorphism, geochemical signatures of meteoritic material, such as enrichments in Platinum Group Elements (PGEs), can be accepted as evidence of impact^26–28^. These highly siderophile elements are heavily sequestered in the core and have low abundance in crustal rocks but have high concentrations in most extraterrestrial materials. However, the presence of an anomaly in just one PGE alone is not enough to uniquely identify an impact origin. While PGE concentrations in surficial rocks typically are in the parts per trillion (ppt) range, environments exist on Earth where Pt and/or Ir concentrations can reach several parts per billion (ppb) such as in deep-deposited organic shales, surficial biomass deposits^29^, mantle xenoliths or deep-sourced volcanic rocks, and in some slags derived from industrial processing of ores. Therefore, a broader geologic context is necessary for interpretations. To convincingly show evidence of meteoritic components, the full suite of PGEs should match known meteorite groups ^28^. The PGE data presented here is limited to only Pt and Ir, which is not sufficient to indicate impact origin. Additionally, PGE concentrations can be notoriously challenging to measure^28–30^ unless using either Instrumental Neutron Activation Analysis (INNA), or highly standardized (fire assay) plasma mass spectrometry. Bunch et al.^1^ use electron microscopy energy dispersive spectrometry (EDS), which has been shown to be inadequate and overestimates Pt abundances by up to a factor of 4^30^. This is particularly problematic at low accelerating voltages, and at the 10 kV voltage used in this study, there is not enough energy to separate the PGE peaks in EDS.

In summary, we feel that the observations and data presented here do not meet the well-established criteria for documenting evidence of an extraterrestrial event. The limited quartz grains presented do not contain convincing of a hypervelocity shock. Claims of ultra-high temperature melt formation and meteoritic geochemistry have not been uniquely demonstrated to be the product of exogenic process. Recognizing impacts and air burst events in the recent geologic record, however, is an important component of planetary defense, and understanding the interaction between these events and civilizations is an exciting and critical aspect of the field. This work does, however, demonstrate the difficulty of relying upon mineralogical and geochemical criteria that can be significantly affected by human technology when analyzing materials in the time period after the development of human-initiated industrial and ritual based melting.
